# Supplementary material for: Comprehensive Comparison of RNA-Seq Data of SARS-CoV-2, SARS-CoV and MERS-CoV Infections: Alternative Entry Routes and Innate Immune Responses
Source: Front Immunol. 2021 May 28;12:656433. doi: 10.3389/fimmu.2021.656433 (PMC8195239; doi:10.3389/fimmu.2021.656433)
Supplement: Supplementary file 1 [file DataSheet_1.pdf]

Supplementary

Yingying Cao[1*], Xintian Xu[2], Simo Kitanovski[1], Lina Song[3], Jun Wang[1], Pei Hao[2,4*], Daniel Hoffmann[1*]

[1]Bioinformatics and Computational Biophysics,
Faculty of Biology and Center for Medical Biotechnology,
University of Duisburg-Essen, Essen 45141, Germany
[2]Key Laboratory of Molecular Virology and Immunology,
Institut Pasteur of Shanghai, Center for Biosafety Mega-Science,
Chinese Academy of Sciences, Shanghai 200031, China
[3]Translational Skin Cancer Research,
German Consortium for Translational Cancer Research, Essen, Germany
[4]The Joint Program in Infection and Immunity:
a. Guangzhou Women and Children's Medical Center,
Guangzhou Medical University, Guangzhou 510623, China;
b. Institut Pasteur of Shanghai,
Chinese Academy of Sciences, Shanghai 200031, China

*To whom correspondence should be addressed;
E-mail: daniel.hoffmann@uni-due.de, phao@ips.ac.cn, yingying.cao@uni-due.de.

1 Additional information about public data

All data can be downloaded from public repositories, the three main sources are NCBI (*1*) (https://www.ncbi.nlm.nih.gov/) and ENA (*2*) (https://www.ebi.ac.uk/ena) and BIG Data Center (*3*) (https://bigd.big.ac.cn/).

1.1 GSE147507 dataset (*4*)

From this dataset we downloaded: Biological triplicates of primary human lung epithelium (NHBE) which were mock treated or infected with SARS-CoV-2 (USA-WA1/2020) at an MOI

of 2; Biological triplicates of transformed lung alveolar (A549) cells which were mock treated or infected with SARS-CoV-2 (USA-WA1/2020) at an MOI of 0.2 or 2; Biological triplicates of transformed lung alveolar (A549) transduced with a vector expressing human ACE2, which were also mock treated or infected with SARS-CoV-2 (USA-WA1/2020) at an MOI of 0.2 or 2; Biological triplicates of transformed lung-derived Calu-3 cells which were mock treated or infected with SARS-CoV-2 (USA-WA1/2020) at an MOI of 2; COVID-19 patient samples: Uninfected human lung biopsies derived from one male (age 72) and one female (age 60) and used as control biological replicates, and lung samples derived from a single male COVID-19 deceased patient (age 74) which were processed in technical replicates. Library preparation method polyA+ selection was used to remove rRNAs before sequencing.

1.2 GSE148729 dataset (*5*)

From this dataset we downloaded biological replicates of Calu-3, Caco-2 and H1299 cells which were mock treated or infected with SARS-CoV-2 (patient isolate BetaCoV/Munich/BavPat1/2020/ EPI_ISL_406862) or SARS-CoV (Frankfurt strain) at an MOI of 0.3. Library preparation method polyA+ selection was used to remove rRNAs before sequencing Caco-2 and H1299 cells. For Calu-3 cells, two library preparation method polyA+ selection and rRNA-depletion were used respectively to remove rRNAs before sequencing.

1.3 GSE153940 dataset

From this dataset we downloaded RNA sequencing data of Vero E6 cells which were either mock-infected or infected with SARS-CoV-2 USA-WA1/2020 (MOI = 0.3) with three replicates. However, when we downloaded the data one sample with accession number GSM4658806 was not available for downloading. Cells were harvested at 24 hours after infection, and rRNA-depletion method was used to extract RNA for sequencing.

1.4 GSE56189 dataset

From this dataset we downloaded: Biological triplicates of MRC5 and Vero E6 cells which were mock treated or infected with SARS-CoV (Urbani strain) or MERS-CoV (EMC/2012) at an MOI of 0.1 or 3. Library preparation method polyA+ selection was used to remove rRNAs before sequencing.

1.5 CRA002390 dataset (*6*)

This dataset is public available in https://bigd.big.ac.cn/gsa/browse/CRA002390. From this dataset we downloaded: The raw FASTQ data of PBMC and BALF samples of COVID-19 patients and corresponding PBMC controls.

1.6 PRJNA434133 dataset (*7*)

From this dataset we downloaded the raw FASTQ data for BALF healthy control samples with accession numbers SRR10571724, SRR10571730, and SRR10571732.

1.7 GSE145926 dataset (*8*)

From this dataset we downloaded the preprocessed single cell RNA-Seq data of BALF samples from 6 severe COVID-19 patients and 3 mild COVID-19 patients.

1.8 PRJNA526088 dataset (*9*)

From this dataset we downloaded the preprocessed single cell RNA-Seq data of BALF sample from a healthy control with accession number GSM3660650.

2 Supplementary figures

Fig. S1:

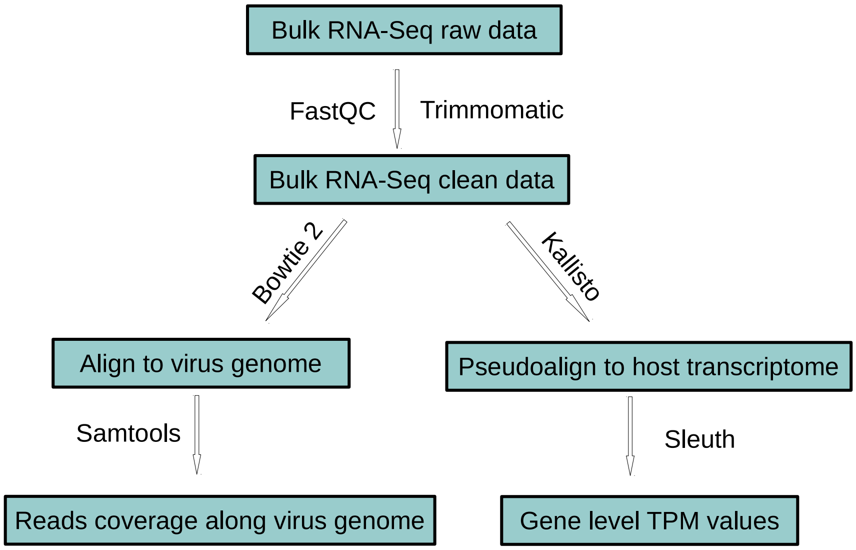

Fig. S2:

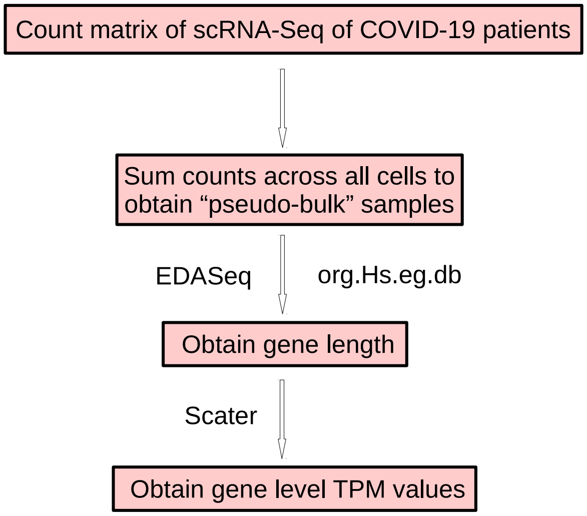

Fig. S3:

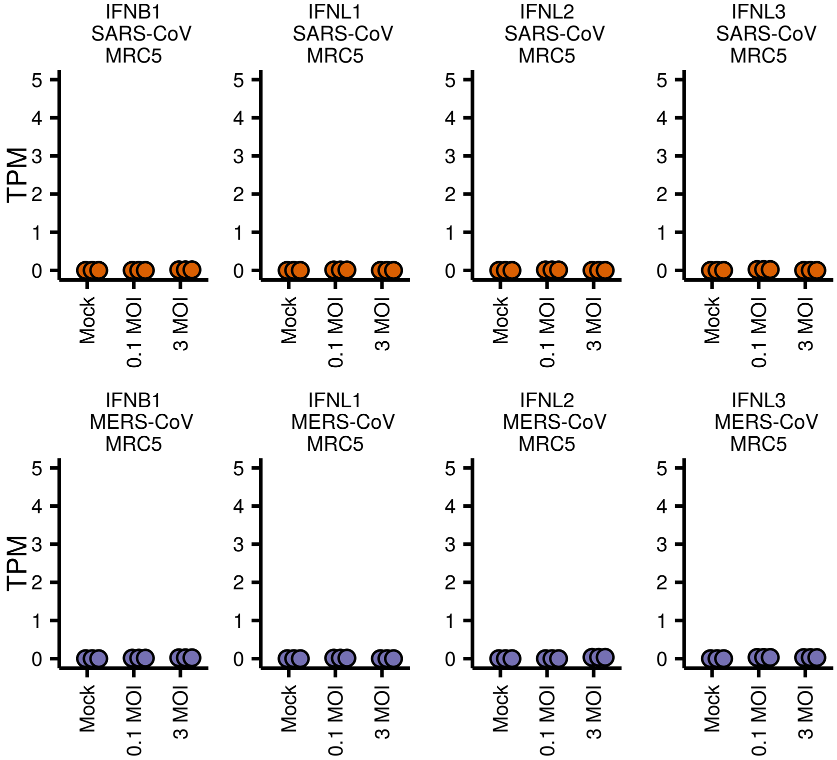

IFNB1 SARS-CoV MRC5
IFNL1 SARS-CoV MRC5
IFNL2 SARS-CoV MRC5
IFNL3 SARS-CoV MRC5
IFNB1 MERS-CoV MRC5
IFNL1 MERS-CoV MRC5
IFNL2 MERS-CoV MRC5
IFNL3 MERS-CoV MRC5
TPM
0
1
2
3
4
5
Mock
0.1 MOI
3 MOI

Fig. S4:

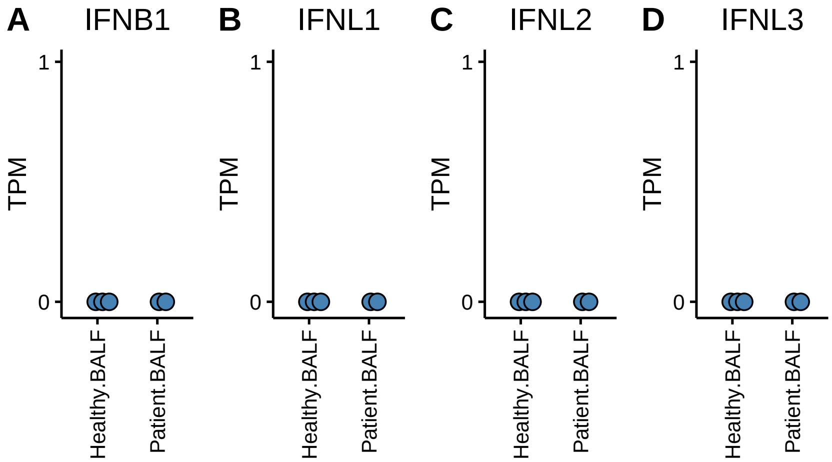

A
IFNB1
B
IFNL1
C
IFNL2
D
IFNL3
TPM
1
0
Healthy.BALF
Patient.BALF

Fig. S5:

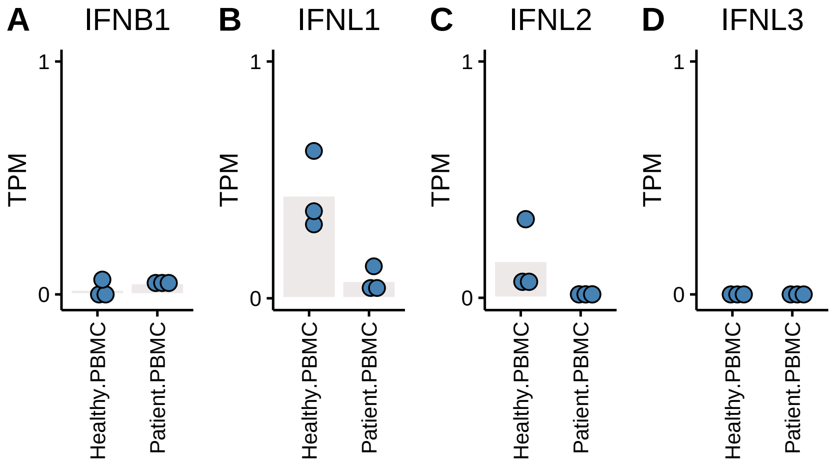

A
IFNB1
B
IFNL1
C
IFNL2
D
IFNL3
1
0
TPM
Healthy.PBMC
Patient.PBMC

Fig. S6:

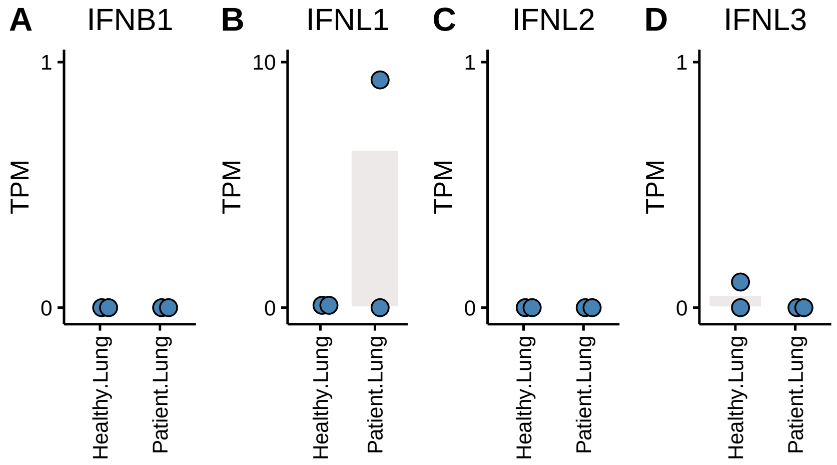

A
IFNB1
B
IFNL1
C
IFNL2
D
IFNL3
TPM
1
0
10
Healthy.Lung
Patient.Lung

Fig. S7:

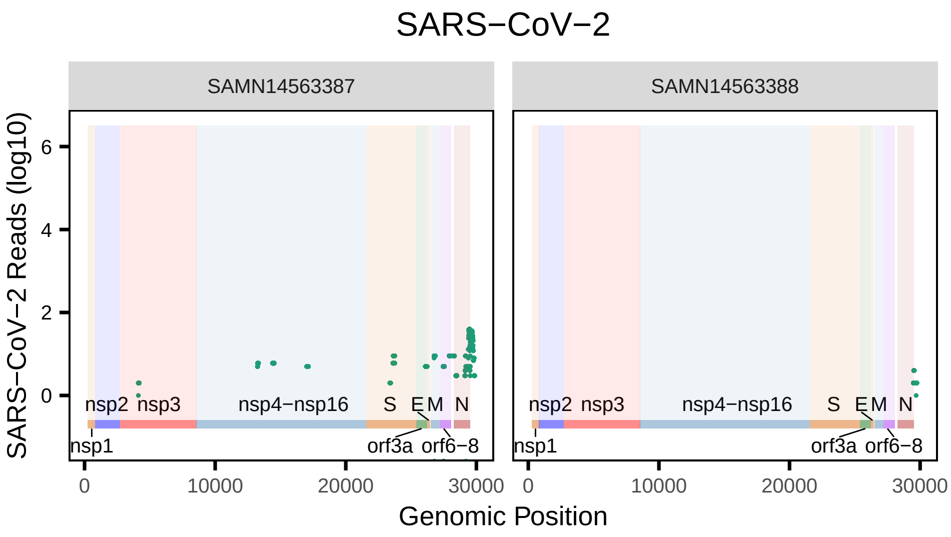
SARS−CoV−2
SAMN14563387
SAMN14563388
SARS−CoV−2 Reads (log10)
0
2
4
6
nsp1
nsp2
nsp3
nsp4−nsp16
S
E
M
N
orf3a
orf6−8
0
10000
20000
30000
Genomic Position

Fig. S8:

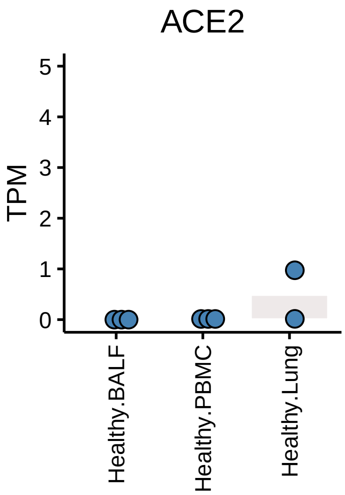

References

1. D. L. Wheeler, T. Barrett, D. A. Benson, S. H. Bryant, K. Canese, V. Chetvernin, D. M. Church, M. DiCuccio, R. Edgar, S. Federhen, *et al.*, Database resources of the national center for biotechnology information. *Nucleic acids research* **36**, D13–D21 (2007).

2. R. Leinonen, R. Akhtar, E. Birney, L. Bower, A. Cerdeno-Tárraga, Y. Cheng, I. Cleland, N. Faruque, N. Goodgame, R. Gibson, *et al.*, The european nucleotide archive. *Nucleic acids research* **39**, D28–D31 (2010).

3. N. G. D. C. Members, *et al.*, Database resources of the national genomics data center in 2020. *Nucleic Acids Research* **48**, D24 (2020).

4. D. Blanco-Melo, B. E. Nilsson-Payant, W.-C. Liu, S. Uhl, D. Hoagland, R. Møller, T. X. Jordan, K. Oishi, M. Panis, D. Sachs, *et al.*, Imbalanced host response to sars-cov-2 drives development of covid-19. *Cell* (2020).

5. E. Wyler, K. Mösbauer, V. Franke, A. Diag, L. T. Gottula, R. Arsie, F. Klironomos, D. Koppstein, S. Ayoub, C. Buccitelli, *et al.*, Bulk and single-cell gene expression profiling of sars-cov-2 infected human cell lines identifies molecular targets for therapeutic intervention. *bioRxiv* (2020).

6. Y. Xiong, Y. Liu, L. Cao, D. Wang, M. Guo, A. Jiang, D. Guo, W. Hu, J. Yang, Z. Tang, *et al.*, Transcriptomic characteristics of bronchoalveolar lavage fluid and peripheral blood mononuclear cells in covid-19 patients. *Emerging microbes & infections* **9**, 761–770 (2020).

7. D. Michalovich, N. Rodriguez-Perez, S. Smolinska, M. Pirozynski, D. Mayhew, S. Uddin, S. Van Horn, M. Sokolowska, C. Altunbulakli, A. Eljaszewicz, *et al.*, Obesity and disease severity magnify disturbed microbiome-immune interactions in asthma patients. *Nature communications* **10**, 1–14 (2019).

8. M. Liao, Y. Liu, J. Yuan, Y. Wen, G. Xu, J. Zhao, L. Cheng, J. Li, X. Wang, F. Wang, *et al.*, Single-cell landscape of bronchoalveolar immune cells in patients with covid-19. *Nature medicine* pp. 1–3 (2020).

9. C. Morse, T. Tabib, J. Sembrat, K. L. Buschur, H. T. Bittar, E. Valenzi, Y. Jiang, D. J. Kass, K. Gibson, W. Chen, *et al.*, Proliferating spp1/mertk-expressing macrophages in idiopathic pulmonary fibrosis. *European Respiratory Journal* **54** (2019).
